# Circulating metabolite profiles to predict response to cardiac resynchronization therapy

**DOI:** 10.1186/s12872-020-01443-y

**Published:** 2020-04-16

**Authors:** Xue Gong, Zhonghan Sun, Zheyong Huang, Qian Zhou, Ziqing Yu, Xueying Chen, Wenqi Shao, Yan Zheng, Yixiu Liang, Shengmei Qin, Yangang Su, Junbo Ge

**Affiliations:** 1 Department of Cardiology, Shanghai Institute of Cardiovascular Disease, Zhongshan Hospital, Fudan University, Shanghai, 200032 People’s Republic of China; 2 Human Phenome Institute, Fudan University, Shanghai, 200438 People’s Republic of China; 3 Department of Traditional Chinese Medicine, Xijing Hospital, Fourth Military Medical University, Xi’an, 710032 People’s Republic of China; 4 Department of Laboratory, Zhongshan Hospital, Fudan University, Shanghai, 200032 People’s Republic of China

**Keywords:** Heart failure, Metabolism, Cardiac resynchronization therapy

## Abstract

**Background:**

Heart failure is associated with ventricular dyssynchrony and energetic inefficiency, which can be alleviated by cardiac resynchronization therapy (CRT) with approximately one-third of non-response rate. Thus far, there is no specific biomarker to predict the response to CRT in patients with heart failure. In this study, we assessed the role of the blood metabolomic profile in predicting the response to CRT.

**Methods:**

A total of 105 dilated cardiomyopathy patients with severe heart failure who received CRT were included in our two-stage study. Baseline blood samples were collected prior to CRT implantation. The response to CRT was defined according to echocardiographic criteria. Metabolomic profiling of serum samples was carried out using ultrahigh performance liquid chromatography coupled with quadrupole-time-of-flight mass spectrometry.

**Results:**

Seventeen metabolites showed significant differences in their levels between responders and non-responders, and these metabolites were primarily involved in six pathways, including linoleic acid metabolism, Valine, leucine and isoleucine biosynthesis, phenylalanine metabolism, citrate cycle, tryptophan metabolism, and sphingolipid metabolism. A combination of isoleucine, tryptophan, and linoleic acid was identified as an ideal metabolite panel to distinguish responders from non-responders in the discovery set (*n* = 51 with an AUC of 0.981), and it was confirmed in the validation set (*n* = 54 with an AUC of 0.929).

**Conclusions:**

Mass spectrometry based serum metabolomics approach provided larger coverage of metabolome which can help distinguish CRT responders from non-responders. A combination of isoleucine, tryptophan, and linoleic acid may associate with significant prognostic values for CRT.

## Background

Cardiac resynchronization therapy (CRT) has been shown to be efficacious to improve cardiac function and become a standard treatment for patients with severe heart failure (HF) [1]. However, approximately one-third of the patients did not obtain any benefit from this therapy [2], and the mechanism remains unclear. To date, a limited number of studies have focused on finding specific biomarkers to predict the response to CRT. Myocardial imaging techniques assessing myocardial mechanical activation time and dyssynchrony are somewhat limited by low sensitivity and large observer variability to predict response to CRT [3]. The evaluation of electrical dyssynchrony using QRS duration (QRSd) was more reliable to predict CRT outcome [4]. Nonetheless, in patients with QRSd < 150 ms, there are limited data on markers of electrical dyssynchrony [5]. Hence, recognition of patients who are likely to benefit from CRT before the device implantation is still challenging.

As an alternative strategy for biomarker discovery, metabolomics based on nuclear magnetic resonance (NMR) and mass spectrometry (MS) enables to identify the endogenous small-molecule metabolites that are sensitively associated with pathological alterations [6], which is believed to have the potential to provide individualized and predictive information for disease progression and personalization of specific medical treatment [7, 8]. Up to now, two published reports investigated the metabolite changes, contributing toward predicting response to CRT. In a study conducted based on 1H-NMR technique, the accuracy of discrimination between responders and non-responders remained low [9]. In another report based on a wide range of targeted metabolite profiling with the use of gas chromatography-mass spectrometry (GC-MS) and 1H-NMR techniques, it was revealed that CRT responders may have a favorable metabolomic profile as a potential biomarker for predicting CRT outcome [10]. It is also reported that the sensitivity of 1H NMR is relatively low when compared with MS [11]. Therefore, it is meaningful to apply complementary liquid chromatography-mass spectrometry (LC-MS) based metabolomics platforms to identify novel biomarkers to predict CRT outcome.

In this study, we conducted a two-stage study to examine the role of the serum metabolite profile based on ultrahigh performance LC coupled with quadrupole-time-of-flight-MS (UHPLC-Q-TOFMS) to predict the response to CRT.

## Methods

### Study subjects

In this study, a total of 105 dilated cardiomyopathy (DCM) patients with severe HF who received CRT were included. Primary DCM was diagnosed based on the conventional criteria of a left ventricular ejection fraction (LVEF) of less than 45%, a dilated left ventricle (an end diastolic diameter of greater than 2.7 cm/m^2^) and unknown causes of myocardial disease [12]. Accordingly, patients with various secondary causes of HF, such as ischemic cardiomyopathy and valvular heart disease were excluded to diminish the potential confounding effects of etiological heterogeneity of HF on CRT. All subjects were admitted to Zhongshan Hospital, Fudan University (Shanghai, China) between March 2014 and December 2016.

According to echocardiographic criteria, patients who had an increase of LVEF ≥5% after 6 months of CRT treatment was defined as responders to CRT [13], patients with an increase of LVEF < 5% were categorized as non-responders. In current study, subjects were divided into discovery set (*n* = 51, 27 responders and 24 non-responders) and validation set (*n* = 54, 36 responders and 18 non-responders). Baseline blood samples from 105 subjects were collected prior to CRT implantation. The collected blood samples were clotted for 45 min at room temperature and centrifuged for 10 min at 3000 rpm; the upper serum phase was then isolated, aliquoted and frozen at − 80 °C until analysis. Demographic characteristics and clinical data were obtained from electronic medical record review. The study protocol was reviewed and approved by the Ethics Committee of Zhongshan Hospital, Fudan University. All patients signed written informed consent form as well.

### Metabolite profiling based on UHPLC-Q-TOFMS

A global serum metabolic profiling based on UHPLC-Q-TOFMS was conducted with 100ul serum samples by using Agilent 1290 Infinity LC system (Agilent Technologies, Santa Clara, CA, USA) coupled with an Agilent 6530 Accurate-Mass-Q-TOF mass spectrometer (Agilent Technologies, Santa Clara, CA, USA). Quality control (QC) samples were aliquots of a pooled sample of the whole sample set and were evenly distributed in real sequence to assess the stability of the LC-MS technique [14]. All analyses were acquired using a mixture of 10 mM purine and 2 mM hexakis phosphazene as internal standards to ensure mass accuracy and reproducibility. The other detailed LC–MS protocols could be found from a previously published paper [15]. Metabolite peaks were identified by referring to open-access databases METLIN (http://metlin.scripps.edu) and HMDB (http://www.hmdb.ca) or related literature within a mass accuracy of 30 ppm.

### Statistical analysis

Differences of demographic characteristics and clinical data between responders and non-responders were analyzed by independent-samples t-test for continuous variables and χ^2^ test or Mann-Whitney U test for categorical variables. A *P*-value of less than 0.05 was considered statistically significant.

Principal component analysis (PCA) and orthogonal projection to latent structures-discriminant analysis (OPLS-DA) were to study metabolic differences between the different group of samples. The statistical significances for metabolites’ relative intensities between responder group and non-responder group were calculated using the independent-samples t-test. The variable importance in projection (VIP) values of all peaks was also generated to be a coefficient for peak selection. Features with VIP > 1.0 and *P* < 0.05 between the two groups were regarded as potential biomarkers and selected for identification. The risk of overfitting was evaluated using R^2^ and Q^2^ values from 99 times permutation in the OPLS-DA model [16].

Receiver operating characteristics (ROC) were generated, and the area under the ROC curve (AUC), sensitivity and specificity were calculated to assess the predictive ability of metabolite biomarkers. To achieve a better predictive ability with multiple metabolites, logistic regression analysis with stepwise selection was employed to perform selection of potential biomarkers, and a metabolite panel was established as the weight sum of metabolite with the beta coefficient as the weight. All the above analyses were conducted using SMICA-P software (version 11.0, Umetrics) and R (version 3.5.1, https://www.r-project.org/).

To identify the differential metabolic pathways between non-responder and responder groups, the metabolic pathway analysis was performed by the MetaboAnalyst 4.0 based on the differential metabolites [17].

## Results

### Demographic and clinical data between the two groups before CRT implantation

Baseline demographic characteristics and clinical data of 105 DCM patients were presented in Table 1. Age, sex, BMI, blood biochemical indices and medication use of patients with different response to CRT showed no significant difference in both discovery set and validation set. When pooling discovery set and validation set together, QRS duration was significantly longer in responders than non-responders (*P*-value < 0.01, data not shown).

**Table 1:** Demographic and clinical data between the two groups before CRT implantation

	Discovery set		Validation set	
	Responders	Non-responders	Responders	Non-responders
	*n* = 27	*n* = 24	*n* = 36	*n* = 18
Female (%)	10 (37%)	6 (25%)	13 (36.1%)	6 (33.3%)
Age (years)	64.30 ± 10.94	62.33 ± 11.56	61.69 ± 10.85	63.89 ± 7.13
BMI (kg/m^2^)	22.91 ± 2.24	23.81 ± 1.91	23.05 ± 3.35	21.86 ± 2.97
NYHA class (II/III/IV) (n)	9/19/2	6/14/4	7/26/3	3/12/3
LVEF (%)	30.48 ± 5.22	29.79 ± 5.01	28.92 ± 6.33	27.28 ± 6.09
LVEDD (mm)	69.78 ± 7.86	73.54 ± 10.82	69.64 ± 6.94	73.50 ± 8.08
QRS duration (ms)	159.81 ± 19.72	149.71 ± 19.41	161.25 ± 16.64	152.67 ± 15.87
CAD (n, %)	5/22 (18.5%)	2/22 (8.3%)	1/35 (2.8%)	0/18 (0.0%)
Hypertension (n, %)	11/16 (40.7%)	7/17 (29.2%)	16/20 (44.4%)	6/12 (33.3%)
Diabetes (n, %)	7/20 (25.9%)	3/21 (12.5%)	8/28 (22.2%)	6/12 (33.3%)
CRT-D (n, %)	15/12 (55.6%)	15/9 (62.5%)	22/14 (61.1%)	11/7 (61.1%)
**Laboratory**				
Log (NT-pro BNP)	3.47 ± 0.52	3.48 ± 0.46	3.29 ± 0.45	3.48 ± 0.48
hs-CRP (mg/L)	11.80 ± 9.74	12.10 ± 13.23	8.58 ± 7.57	12.03 ± 7.77
Creatinine (md/dl)	90.48 ± 36.68	98.46 ± 34.09	82.57 ± 23.55	91.69 ± 18.60
eGFR (ml/min/1.73m^2^)	74.11 ± 23.22	68.63 ± 21.36	80.46 ± 19.66	71.07 ± 16.15
**Medications**				
ACEI/ARB (n, %)	27/0 (100%)	23/1 (95.8%)	32/4 (88.9%)	15/3 (83.3%)
β-blockers (n, %)	27/0 (100%)	22/2 (91.7%)	32/4 (88.9%)	17/1 (94.4%)
MRA (n, %)	27/0 (100%)	22/2 (91.7%)	35/1 (97.2%)	16/2 (88.9%)
Diuretics (n, %)	27/0 (100%)	22/2 (91.7%)	32/4 (88.9%)	18/0 (100%)
Digoxin (n, %)	4/23 (14.8%)	8/16 (33.3%)	19/17 (52.8%)	7/11 (38.9%)
Statins (n, %)	8/19 (29.6%)	4/20 (16.7%)	9/27 (25.0%)	4/14 (22.2%)

***Abbreviation***: *NYHA* New York Heart Association, *LVEF* Left ventricular ejection fraction, *LVEDD* Left ventricular end-diastolic dimension, *CAD* Coronary artery disease, *CRT-D* Cardiac resynchronization therapy- defibrillation, *NT-pro BNP* N-terminal pro-brain natriuretic peptide, *eGFR* estimated glomerular filtration rate, *ACEI/ARB* Angiotensin-converting enzyme inhibitor or angiotensin receptor blocker, *MRA* Aldosterone antagonists. Values are presented as n (%) or mean ± SD. No variable was significantly different between responders and non-responders (all *P* > 0.05)

### Data quality assessment of metabolite profiling

**Fig. 1:**
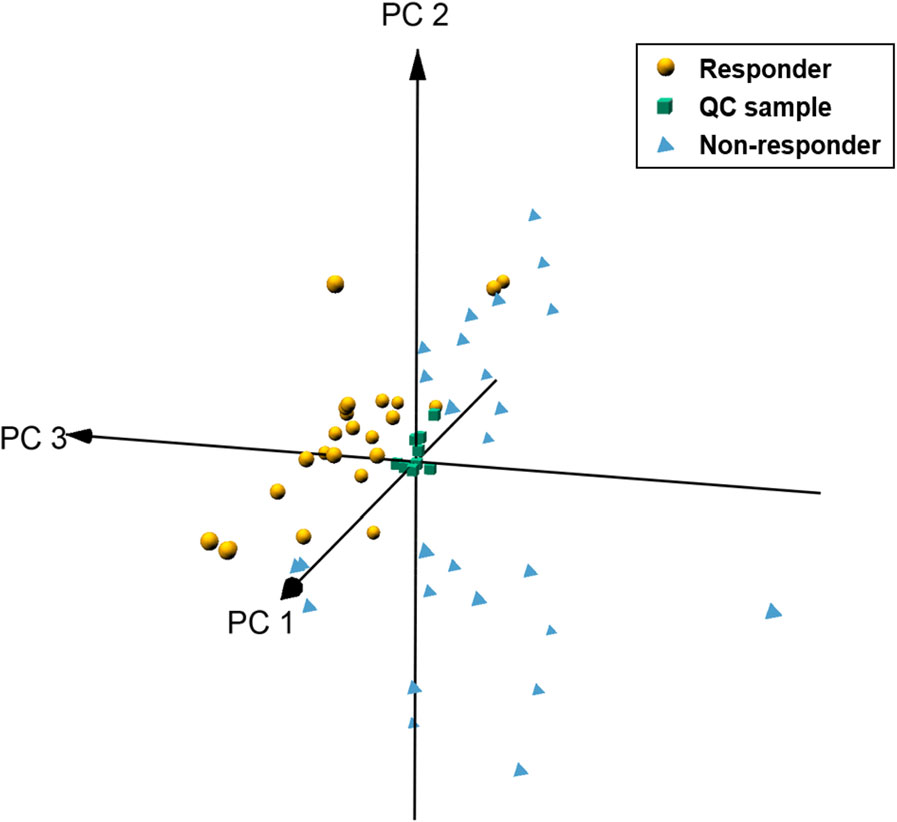
Three-dimensional PCA score plot of discovery set samples and QC samples. Yellow circles, blue triangles, green squares represent responders to CRT, non-responders to CRT and QC samples, respectively

A total of 1284 metabolites was detected in serum samples of CRT patients. After quality control analyses, 1108 metabolites were observed in more than 80% QC samples with relative standard deviations less than 30% in QC samples, covering 86.4% of features in UHPLC-Q-TOF-MS analysis. In addition, three-dimensional PCA score plot was used to provide a visualizable result of the real samples from the discovery set and QC samples after unit variance scaling. As shown in Fig. 1, the close clustering of QC samples was observed, reflecting that the present method had satisfactory repeatability and stability.

### Metabolite differences between non-responder and responder groups

**Fig. 2:**
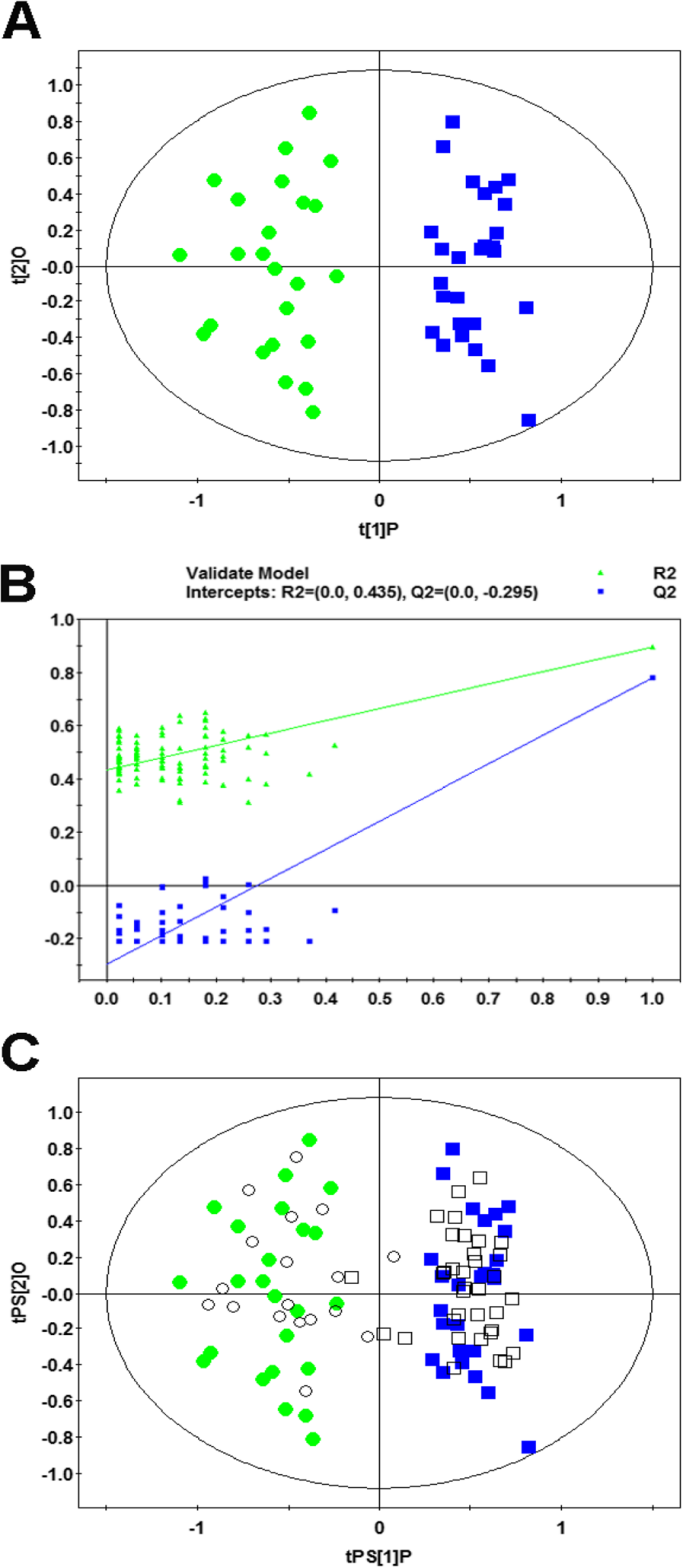
OPLS-DA analysis. **a** OPLS-DA score plot of the discovery set samples. **b** Validation plot of the model obtained from 99 permutation tests. **c** T-predicted scatter plots of the OPLS-DA model. Solid circles and squares represent non-responders and responders to CRT in the discovery set; hollow circles and squares represent non-responders and responders to CRT in the validation set

The three-dimensional PCA score plots showed a tendency of dispersion between non-responder and responder groups in the discovery set (Fig. 1). The OPLS-DA analysis was conducted to further detect the metabolite differences between different groups in the discovery set. As illustrated in Fig. 2a, clearly separations between non-responder and responder groups were observed in the OPLS-DA score plot. After a permutation test repeated 99 times, the R^2^ and Q^2^ of the OPLS-DA model was 0.435 and − 0.295, indicating the model was well fitted and had a reliable predictive ability (Fig. 2b). A validation set (18 non-responders and 36 responders) was classified using the OPLS-DA model to validate the detection ability of this model for response to CRT. As a result, only 2 out of 54 samples were wrongly assigned in the direction of the first principal component, a total of 17 metabolites had significant different serum levels (VIP > 1.0 and *P* < 0.05) between responder and non-responder groups in the discovery set (Table 2). Compared with non-responders, responders exhibited higher levels in valine, citric acid, isoleucine, phenylalanine, indoleacetic acid, lysoPC (14:0), lysoPC (20:3), lysoPC (22:6), and linoleic acid, while lower levels in hypoxanthine, inosine, tryptophan, sphingosine 1-phosphate, hexanoylcarnitine, tetradecenoylcarnitine, linoleyl carnitine, and oleoylcarnitine.

**Table 2:** Differential metabolites for discrimination before CRT in Responders and Non-responders

Metabolite	VIP^a^	*P*-value^b^	AUC^c^	Sensitivity (%)	Specificity (%)
Valine	5.32	8.2E-03	0.70	0.67	0.75
Citric acid	1.07	2.7E-02	0.68	0.78	0.50
Hypoxanthine	2.34	1.1E-02	0.71	0.63	0.75
Isoleucine	15.39	9.1E-06	0.83	0.67	0.83
Inosine	1.03	1.1E-02	0.69	0.63	0.70
Phenylalanine	10.32	1.1E-04	0.79	0.70	0.79
Indoleacetic acid	1.04	2.3E-03	0.75	0.81	0.71
Tryptophan	7.19	2.5E-06	0.84	0.77	0.79
Hexanoylcarnitine	1.01	3.5E-03	0.74	0.63	0.79
Sphingosine 1-phosphate	1.09	3.3E-04	0.77	0.70	0.77
Tetradecenoylcarnitine	0.85	7.4E-04	0.75	0.66	0.75
LysoPC(14:0)	1.59	6.8E-04	0.76	0.62	0.77
Linoleyl carnitine	2.02	4.0E-04	0.76	0.77	0.70
LysoPC(20:3)	3.47	3.8E-03	0.75	0.85	0.62
LysoPC(22:6)	1.31	4.7E-04	0.76	0.77	0.62
Oleoylcarnitine	2.28	6.4E-04	0.78	0.66	0.79
Linoleic acid	1.07	1.8E-03	0.76	0.70	0.75

^a^ Variable importance in the projection (VIP) was obtained from the OPLS-DA model. ^b^*P*-value was calculated from the Student’s t-test. ^c^ Area under the receiver operating characteristic (ROC) curve

### A metabolite panel for CRT response

**Fig. 3:**
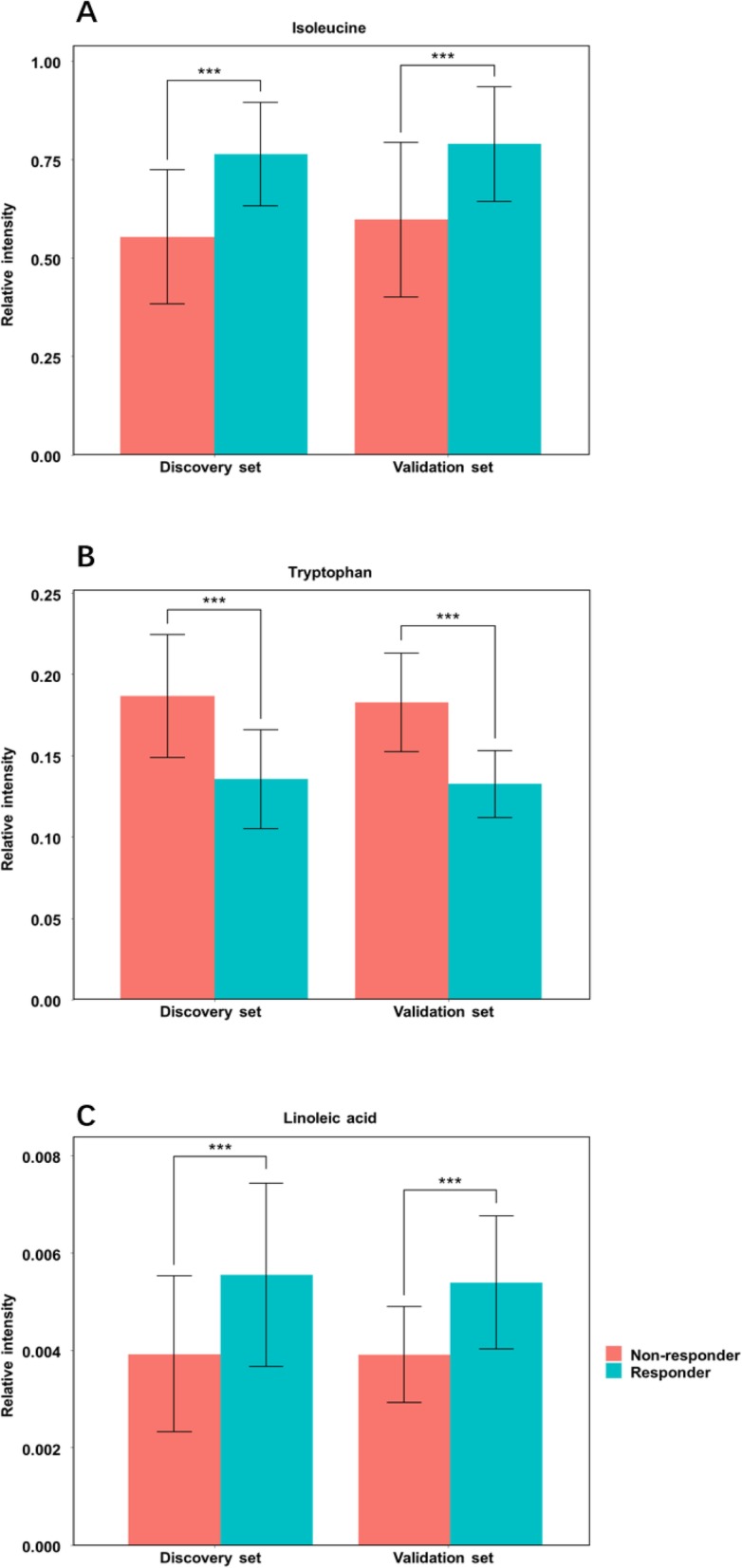
Serum relative concentrations of defined potential biomarkers of Isoleucine (**a**), Tryptophan (**b**) and Linoleic acid (**c**) in the discovery and validation sets. Orange bars represent non-responders to CRT; blue bars represent responders to CRT; and *** represent *P* < 0.001 when compared with non-responder group. All data are expressed as mean ± S.D

**Fig. 4:**
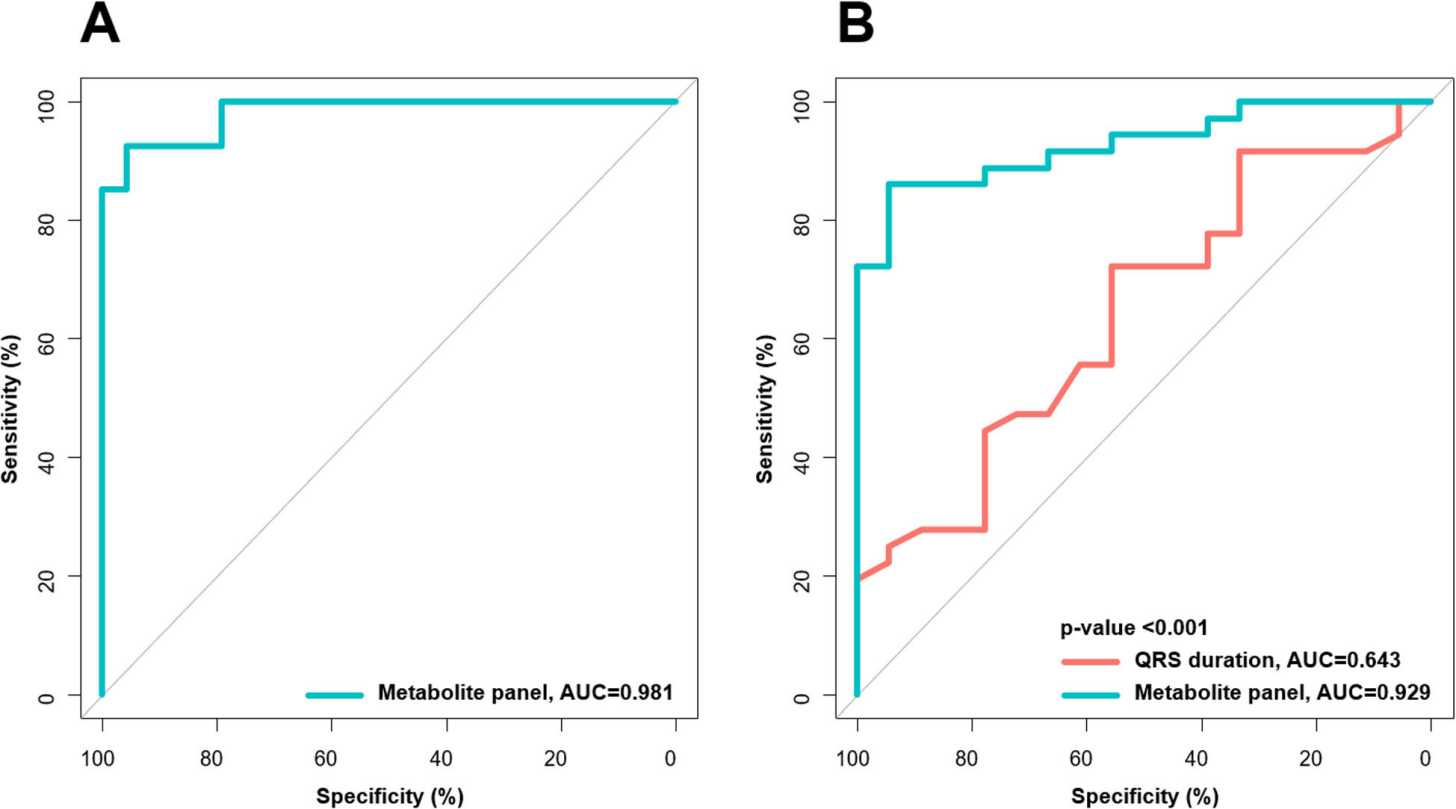
Quantification of the diagnostic performance of the metabolite panel containing Isoleucine, Tryptophan and Linoleic acid in discovery set (**a**) and validation set (**b**). The optimal cutoff value was obtained (0.5276) and applied to evaluate the prediction capacity of the current model. In validation set, QRS duration (orange curve) has an AUC of 0.643 (95% CI: 0.486–0.799) and metabolic panel (blue curve) has an AUC of 0.929 (95% CI: 0.864–0.994)

The values of AUC, sensitivity, and specificity of the top 17 metabolites contributed to the separation between responders and non-responders identified by OPLS-DA were shown in Table 2. Indoleacetic acid and lysoPC (20:3) showed a sensitivity of greater than 0.80 in classifying responders and non-responders, and isoleucine revealed the specificity of greater than 0.80 in classifying responders and non-responders. However, none of these 17 metabolites distinguished responders from non-responders with both sensitivity and specificity of greater than 0.80, which made it necessary to apply multiple serum metabolites in the discrimination of responders out of all HF patients. We therefore established a metabolite panel by using logistic regression analysis together with a stepwise algorithm on the basis of these 17 metabolites. As a result, the combination of isoleucine, tryptophan, and linoleic acid was selected to distinguish responders from non-responders (Fig. 3). The metabolite panel was calculated as the following formula: logit [P = CRT] = 51.04 × [Isoleucine] + 2001.07 × [Linoleic acid] - 94.53 × [Tryptophan] - 27.49, where [P = CRT] represented the detection probability of CRT response based on this metabolite panel, and [Isoleucine], [Linoleic acid], and [Tryptophan] represent the relative serum concentrations. The AUC based on the established model was 0.981 in the discovery set (Fig. 4a), which indicated that the metabolite panel demonstrated an acceptable performance for the prediction of CRT response.

### Validation of the metabolite panel for CRT response

To assess the performance of the selected metabolites for the prediction of CRT response, the AUCs were calculated with QRS duration and metabolite panel in the validation set, respectively. Compared with QRS duration, metabolite panel had a higher AUC of 0.929 in discriminating responders from non-responders, indicating that the metabolite panel significantly improved the predictability (*P* < 0.001, Fig. 4b).

### Differential metabolic pathways between responder and non-responder groups

**Fig. 5:**
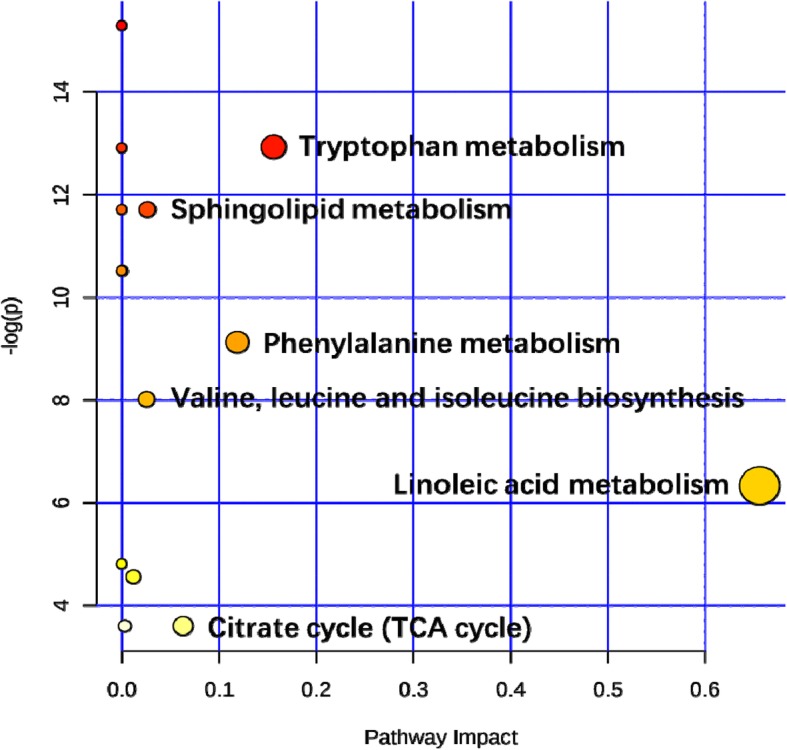
Pathway analysis related to the differential metabolites between non-responder and responder groups. Global metabolite pathways related to the response of CRT were performed by the website of MetaboAnalyst based on all the differential metabolites listed in Table 2. The x-axis represents the pathway impact values from the pathway topology analysis, and the y-axis represents the -log transformation *P* values from the pathway enrichment analysis

A metabolic pathway analysis of all the identified differential metabolites using MetaboAnalyst 4.0 revealed that the metabolic pathways related to CRT response (impact value > 0.02) were mainly associated with linoleic acid metabolism, tryptophan metabolism, phenylalanine metabolism, citrate cycle (TCA cycle), Valine, leucine and isoleucine biosynthesis, and sphingolipid metabolism (Fig. 5).

## Discussion

Using UHPLC-Q-TOFMS-based serum metabolomics approach, we identified a panel of serum metabolites associated with response to CRT in Chinese population. The main findings of this study were as follows: (i) A total of 17 potential metabolites were found to have significant different serum concentrations between CRT responders and non-responders. (ii) Compared with non-responders, responders exhibited higher concentrations in valine, citric acid, isoleucine, phenylalanine, indoleacetic acid, lysoPC, and linoleic acid, while lower concentrations in hypoxanthine, inosine, tryptophan, sphingosine 1-phosphate, and carnitines. (iii) A combination of three serum metabolite biomarkers (isoleucine, tryptophan, and linoleic acid) was established as an ideal metabolite panel to distinguish responders from non-responders.

To understand the underlying molecular functions of these serum metabolite biomarkers, we further conducted metabolic pathway analysis. These 17 metabolites were found to be primarily involved in six metabolism pathways, including linoleic acid metabolism, valine, leucine and isoleucine biosynthesis, phenylalanine metabolism, citrate cycle (TCA cycle), tryptophan metabolism, and sphingolipid metabolism. This metabolic profile covered different aspects of pathogenesis, especially anomalous lipid and energy metabolism. The disturbed metabolic pathways are discussed in detail below.

Linoleic acid is a kind of n-6 polyunsaturated fatty acids and one of the oxidative stress biomarkers [18]. A higher level of linoleic acid could prompt the fatty acid metabolism of myocardial cell, which plays an important role in suppressing cardiomyocyte hypertrophy [19], while advanced left ventricle dilation was reported to be associated with poor CRT outcome [20, 21]. In our study, CRT responders had a lower level of the left ventricular end diastolic dimension and a higher level of linoleic acid, reflecting that linoleic acid might improve the response to CRT by influencing the fatty acid metabolism of myocardial cell.

The high concentrations of valine and isoleucine, correlated with valine, leucine and isoleucine biosynthesis, were observed in CRT responders, which was consistent with Nemutlu et al.’s findings [10]. Valine and isoleucine, two branched-chain amino acids essential for protein synthesis and metabolic signaling, may be important alternative energy substrates. The inhibition of the citrate cycle induced by the HF hypoxia could lead to the utilization of branched-chain amino acids as energy compensation [11, 22]. On the other hand, Colak et al. [23] discovered that genes involved in energy metabolic processes, such as the citric acid cycle and adenosine triphosphate synthesis, were upregulated in DCM patients. The results above heightened that metabolic activity may be a compensatory mechanism in the process of HF. The higher concentration of citric acid in our responder subjects indicated that responders may have a better compensatory adaptation, which might improve the response to CRT.

Phenylalanine is a precursor for tyrosine, including adrenaline and noradrenaline, and higher concentration of phenylalanine is observed in the progress of HF due to stress response to reduced cardiac output [24]. Increased phenylalanine concentration has been identified in cross-sectional studies among individuals with established HF compared with normal controls [24–26], indicating that phenylalanine metabolism may be related to the decline of cardiac function and therefore influences CRT outcomes.

Beyond the metabolites essential for protein synthesis and metabolic signaling [27], we also observed the significant associations between the response to CRT and the following metabolites related to oxidative stress and inflammation. In our study, responders were found to have a lower level of tryptophan, which was reported to be reversely associated with incident cardiovascular disease in a randomized controlled trial [28]. Indoxyl sulfate, a gut bacteria-derived product of tryptophan, could stimulate oxidative stress and, further contributes to the progression of cardiovascular disease, cardiac hypertrophy and fibrosis [29]. The prognostic value of indoxyl sulfate has been proposed for DCM patients with normal renal function or mild-to-moderate chronic kidney diseases [30], and high serum indoxyl sulfate was a significant predictor of cardiac events [31]. Associations between indoxyl sulfate and overall mortality and cardiovascular disease were also reported [31].

Our results showed that CRT responders had a lower level of sphingolipid than non-responders. A recent study revealed that sphingosine and sphinganine levels were decreased in patients with systolic HF due to ischemic or non-ischemic heart disease compared with healthy individuals [32]. Sphingosine 1-phosphate is a bioactive sphingolipid with important functions in immunity, inflammation, and cardiovascular biology [33]. It is associated with the impairment of LVEF, dyspnea, and causally involved in the pathophysiology of HF [33]. Animal models has demonstrated that the deletion of cardiac sphingosine 1-phosphate receptor 1 could lead to the incident of cardiomyopathy and HF [34]. Together with our results, these findings indicated that metabolites might influence the response to CRT via different pathways including energetic metabolism and oxidant stress.

Considering the moderate and unstable predictive ability of individual metabolite, a metabolite panel was constructed using three serum metabolites (isoleucine, tryptophan, and linoleic acid) to distinguish the indication of CRT and showed an ideal capacity to predict the response to CRT. Compared with QRSd, the metabolite panel increased about 50% of the prediction ability, which have a great clinical potential for both doctors and patients to reduce nonessential treatment and to lower medical costs.

This study had several limitations. First, metabolite profiles are variable in vivo because of human activities and changes in the external environment. However, the patients provided blood sample typically a day ahead of or during CRT implantation. In such a short time, the change of metabolite levels could be limited. Second, primary DCM is a genetically heterogeneous disease, and the genetic heterogeneity of the cases is a potential confounder that could influence the serum metabolome. Third, only patients with DCM were investigated in our study, therefore, the generalizability of our conclusion was limited, but this could minimize the impact of the type of disease on metabolite analysis results. Finally, the sample size of the study was relatively small. Consequently, further investigations in larger populations are warranted to explore the corresponding mechanisms of these metabolites related to CRT. Specific factors such as genetic polymorphisms to predict the response of CRT is also worthy of being investigated in the future.

## Conclusions

In conclusion, an UHPLC-Q-TOFMS-based serum metabolomics approach has been developed to profile CRT-related metabolic change in serum. A metabolic set of isoleucine, tryptophan, and linoleic acid is helpful in predicting CRT response. These metabolites, essentially those for energy metabolism, may represent a better metabolic reserve and a higher potential for metabolic recovery in CRT responders.

## Data Availability

The datasets used and/or analyzed during the current study are available from the corresponding author on reasonable request.

## Abbreviations

ACEI/ARB: Angiotensin-converting enzyme inhibitor or angiotensin receptor blocker
AUC: Area under the ROC curve
CAD: Coronary artery disease
CRT(−D): Cardiac resynchronization therapy(−defibrillation)
DCM: Dilated cardiomyopathy
eGFR: Estimated glomerular filtration rate
GC-MS: Gas chromatography-mass spectrometry
HF: Heart failure
HPLC: High performance liquid chromatography
LC-MS: Liquid chromatography-mass spectrometry
LVEDD: Left ventricular end-diastolic dimension
LVEF: Left ventricular ejection fraction
MS: Mass spectrometry
NMR: Nuclear magnetic resonance
NT-pro BNP: N-terminal pro-brain natriuretic peptide
NYHA: New York heart association
OPLS-DA: Orthogonal projection to latent structures-discriminant analysis
QC: Quality control
ROC: Receiver operating characteristics
UHPLC-Q-TOFMS: Ultrahigh performance-LC coupled with quadrupole-time-of-flight-MS
VIP: Variable importance in projection
